# An Unusual Presentation of Ovarian Teratoma: A Case Report

**DOI:** 10.1155/2012/845198

**Published:** 2012-06-03

**Authors:** Seema Khanna, Vivek Srivastava, Sanjai Saroj, Shashi Prakash Mishra, S. K. Gupta

**Affiliations:** Department of General Surgery, Institute of Medical Sciences, Banaras Hindu University, Varanasi 221005, India

## Abstract

Mature cystic teratomas account for 10–20% of all ovarian neoplasms and are the most common neoplasm in younger patients. Spontaneous rupture of the teratoma is rare and has been occasionally reported. Here we present an unusual case of left ovarian teratoma rupturing into sigmoid colon.

## 1. Introduction

Mature cystic teratomas account for 10–20% of all ovarian neoplasms and are the most common neoplasm in patients younger than 20 years of age. Mature teratomas are usually benign, but in 0.1-0.2% of cases, it may undergo malignant transformation [1]. They may remain asymptomatic or may present with acute abdomen because of torsion, infection, or rupture. Spontaneous rupture of the teratoma is rare and has been occasionally reported. Here we present an unusual case of left ovarian teratoma rupturing into sigmoid colon.

## 2. Case Report

An eighteen years old female presented in the emergency OPD with the complaints of pain abdomen, fever, non-passage of flatus and stool, and nonprojectile, nonbilious vomiting of two days duration. There was history of typhoid fever four months back for which she received treatment and recovered. Menarche was at 14 years and menstrual cycles were normal. On examination the abdomen was distended, tense, and tender with marked guarding and rigidity. No other obvious lump or organomegaly was detected. There was masking of liver dullness with evidence of free fluid in peritoneal cavity. Digital rectal examination revealed free fluid in the pouch of Douglas.

Plain X-ray abdomen revealed free gas under the diaphragm. There was mild leucocytosis. In serum biochemistry, blood urea was 60.3 mg/dL and serum creatinine was 1.3 mg/dL.

**Figure 1:**
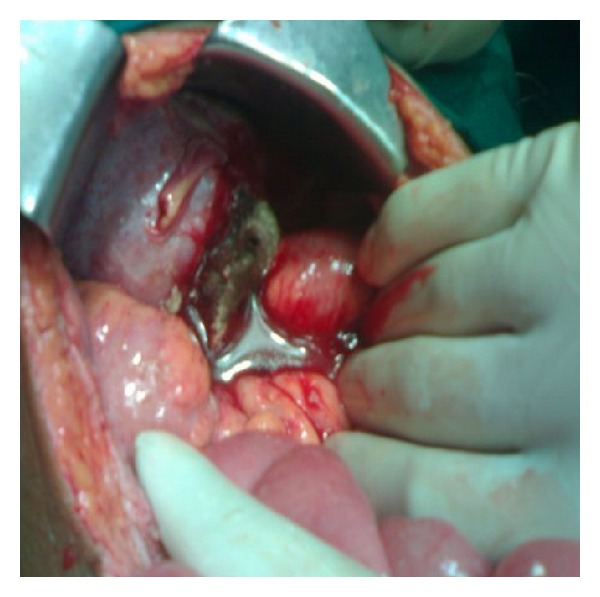
Left ovarian teratoma infiltrating into sigmoid colon.

**Figure 2:**
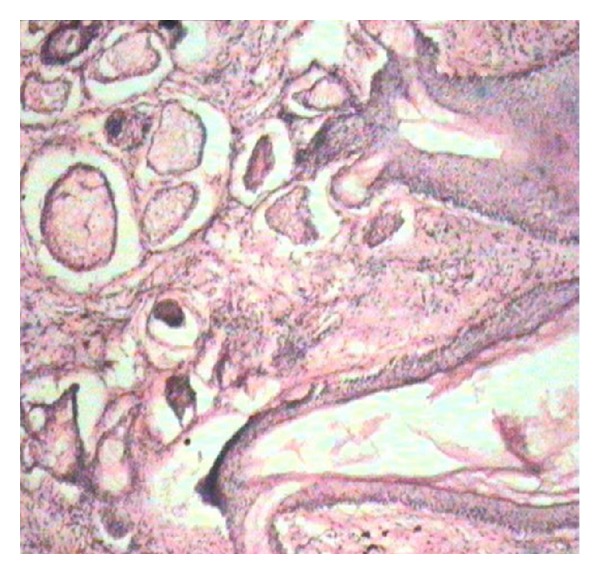
Microphotograph of excised teratoma.

The patient underwent exploratory laparotomy with the diagnosis of perforation peritonitis. On exploration, a large 8 × 8 cm teratoma arising from the left ovary was found infiltrating the sigmoid colon near the rectosigmoid junction leading to perforation of the sigmoid colon (Figure 1). The teratoma consisted of hair and cartilage along with foul-smelling fluid. Left-sided oophorectomy with excision of teratoma was done. The sigmoid colon perforation was closed primarily with proximal defunctioning loop sigmoid colostomy. Histopathology revealed mature dermoid with inflammatory reaction (Figure 2). There was no granulation and no evidence of any malignancy. Postoperatively serum alpha-fetoprotein was 7.2 ng/mL and beta-HCG was 3.7 IU/mL. Postoperative course was uneventful, and reversal of loop colostomy was done after 3 months.

## 3. Discussion

The word “teratoma” is derived from Greek work “teraton” meaning monster. It was initially used by Virchow in 1863. The term “dermoid cyst” was coined by Leblanc in 1831 [2]. Both are used interchangeably.

Arising from totipotential cells, these tumours are midline or paraxial. After sacrococcygeal teratomas (57%), gonads (29%) are the second most common site. Mature cystic teratomas are 10–20% of all ovarian neoplasms, mostly occur in patients less than 20 years of age. Complications of ovarian teratoma include torsion, rupture, infection, and malignant change.

Spontaneous rupture of the cyst is extremely rare because of its thick wall and is reported in 0.3% to 0.7% of cases [1]. The cyst can rupture into the peritoneal cavity or rarely into a hollow abdominal organ. The reported sites are urinary bladder, small bowel rectum, sigmoid colon, and vagina [3]. Colorectal involvement by a benign cystic ovarian teratoma has been reported with varied and unusual presentations. von-Walter and Nelken [4] reported a benign cystic ovarian teratoma with a fistula into the small and large bowel. Awareness of clinical and radiological findings in these cases is important to avoid further complications. Teratomas can invade the rectal wall and can present as a bleeding rectal polyp [5] or can present as a change in bowel habit due to faeces filling the cyst causing compression of rectum [6]. Pararectal teratomas can arise from either the ovaries or the soft tissues in the sacrococcygeal region. Farkouh et al. reported a hyperemic polypoidal mass with a stalk 15 cm from the anal verge on colonoscopy. Computed tomography of this patient revealed a right ovarian mass that was in continuity with the intraluminal lesion in the sigmoid colon [7]. Possible predisposing factors for such presentations include adhesion of the tumor to an adjacent structure resulting in ischemia and necrosis of cyst wall, infection of the cyst, result of trauma during labor, or a malignant change in the dermoid cyst.

Deodhar et al. [8] have reported a retrospective clinicopathological study of 28 cases of immature ovarian teratoma and found immature mesenchyme in all these cases. They concluded that presence of immature mesenchyme in an otherwise mature teratoma should lead to further sampling to search for the presence of neuroepithelium.

In our case, as there was history of typhoid fever four months back, it could have caused inflammation and adhesion of the dermoid cyst to the bowel wall eventually leading to perforation of sigmoid colon.

## 4. Conclusion

 Ovarian teratoma commonly has an indolent course and presents with abdominal pain due to complications like torsion, hemorrhage, or infection. Spontaneous rupture of teratoma is rare due to its thick wall, and it usually occurs in the peritoneal cavity. Rupture into a hollow viscus due to adhesions is extremely rare complication which may present as perforation peritonitis.
